# Role of Endoscopy in Malignant Biliary Obstruction

**DOI:** 10.3390/diagnostics16050721

**Published:** 2026-02-28

**Authors:** Ishaan Vohra, Burraq Imran, Zubair Khan, Muhammad Hasan

**Affiliations:** 1 Department of Medicine, University of Illinois College of Medicine Peoria, Peoria, IL 61605, USA; 2 Carle Methodist Healthcare System, Peoria, IL 61636, USA; 3 University of Exeter Medical School, Exeter EX1 2HZ, UK; 4 AdventHealth, Orlando, FL 32803, USA

**Keywords:** endoscopy, malignant biliary obstruction, endoscopic ultrasound, endoscopic retrograde cholangiopancreatography, transpapillary biliary drainage

## Abstract

Malignant biliary obstruction (MBO) represents a critical clinical challenge characterized by bile duct compromise leading to severe complications, including intractable jaundice, recurrent cholangitis, biliary cirrhosis, and hepatic failure. Classification into distal MBO (DMBO) and hilar MBO (HMBO) guides therapeutic decision-making, with the former predominantly caused by pancreatic head adenocarcinoma and extrahepatic cholangiocarcinoma, while perihilar cholangiocarcinoma represents the principal etiology of the latter. The high morbidity and mortality associated with MBO necessitate prompt, expert intervention. While endoscopic retrograde cholangiopancreatography (ERCP) with transpapillary biliary drainage (TBD) has traditionally served as the cornerstone of management for unresectable tumors, endoscopic ultrasound (EUS)-guided biliary drainage has emerged as a compelling alternative, particularly when conventional ERCP proves technically unsuccessful or anatomically unfeasible. This review comprehensively examines current endoscopic strategies for MBO, emphasizing the complementary roles of ERCP and EUS-based techniques. Optimal outcomes require intervention by experienced endoscopists at high-volume tertiary centers, with individualized treatment selection based on anatomical considerations, tumor characteristics, patient factors, and local expertise.

## 1. Introduction

Malignant biliary obstruction (MBO) constitutes a biliary pathology associated with substantial morbidity and mortality [1,2]. The condition arises from bile duct blockage that compromises normal bile flow, precipitating severe complications such as intractable jaundice, cholangitis, biliary cirrhosis, and ultimately hepatic failure [3,4,5]. MBO is anatomically classified as either distal (DMBO) or hilar (HMBO) based on the obstruction site, though some patients present with multifocal involvement [6].

The majority of DMBO cases result from adenocarcinoma of the pancreatic head and extrahepatic cholangiocarcinoma, with less common etiologies including gallbladder cancer, ampullary neoplasms, lymphoma, and metastatic disease [7,8,9]. Perihilar cholangiocarcinoma (Klatskin tumor), classified using the Bismuth–Corlette system, represents the predominant cause of HMBO. This classification system proves crucial for both interventional planning and resectability assessment. Additional HMBO etiologies include gallbladder carcinoma, hepatocellular carcinoma, lymphoma, and metastatic malignancies [10].

A major clinical challenge lies in the late diagnosis of MBO, particularly for HMBO patients classified as Bismuth–Corlette stage III-IV, who typically present at an inoperable stage. Inoperable disease median survival ranges from 7 to 16 months [10,11], shifting therapeutic goals toward palliation with emphasis on biliary decompression, infection prevention, and optimization for systemic therapy.

Traditionally, transpapillary endoscopic biliary drainage (TBD) via ERCP has served as the gold standard approach regardless of palliative status, offering the advantage of achieving both decompression and tissue diagnosis in a single intervention [12,13]. It is important to note that most EUS-guided biliary drainage procedures are performed following failed ERCP attempts, and ERCP expertise remains more universally available compared to interventional EUS expertise. Therefore, interventional EUS in MBO is an adjunct to conventional ERCP rather than a replacement, only when ERCP is unsuccessful or anatomically infeasible. Ongoing innovation continues to refine endoscopic strategies and broaden their role in comprehensive MBO management.

This review provides a comprehensive overview of current therapeutic strategies for malignant biliary obstruction and critically assesses novel interventions that may expand future clinical options.

## 2. Transpapillary Biliary Drainage

### 2.1. TBD in Unresectable Distal Malignant Biliary Obstruction

Transpapillary biliary drainage with placement of a self-expanding metal stent (SEMS) during ERCP is currently considered the gold-standard approach for decompression of DMBO, achieving technical success rates of 87.5–95% [14]. Anatomical and technical difficulties related to tumor characteristics constitute the most common causes of procedural failure [6,15,16]. TBD demonstrates superiority over surgical bypass owing to lower morbidity and mortality rates, despite higher recurrent biliary obstruction rates [17].

Compared to percutaneous transhepatic biliary drainage (PTBD), TBD offers several advantages, including fewer complications, reduced need for subsequent procedures, shorter hospitalization, and lower costs. Additionally, patients undergoing PTBD experience diminished quality of life and face risks of catheter dislodgement [12,14,18,19,20].

Stent selection remains a critical consideration in DMBO management. Following the introduction of plastic stenting in the 1980s and subsequent emergence of SEMS in the 1990s [21,22], multiple comparative studies have demonstrated SEMS superiority in terms of patency duration, resulting in fewer recurrent obstruction episodes and reduced need for reinterventions [23,24,25]. Despite higher initial costs, an SEMS achieves cost equivalence with plastic stents at one year due to prolonged functional duration [26]. This cost-effectiveness was corroborated by Bor et al., who reported similar cumulative treatment costs between groups, with plastic stents actually incurring higher costs in patients surviving 2–4 months (2888€ vs. 2258€) [27]. However, the SEMS demonstrates a higher incidence of post-ERCP pancreatitis, occurring approximately five times more frequently than with plastic stents [28,29,30,31].

These advantages have led guidelines to recommend SEMS over plastic stents in DMBO. The choice between uncovered SEMS (U-SEMS) and covered SEMS (C-SEMS), which includes partially covered (PC-SEMS) and fully covered (FC-SEMS) variants, presents a more nuanced decision. Each type carries distinct risks: U-SEMS face increased neoplastic ingrowth through metal meshes, while C-SEMS demonstrate higher rates of stent migration, tumor overgrowth, and sludge formation [32,33,34].

Numerous studies comparing C-SEMS with U-SEMS for DMBO have reported no clear superiority regarding patency, safety, or patient survival [35,36,37,38]. However, a retrospective cohort study published in September 2025 found FC-SEMS significantly prolonged stent patency (445 vs. 348 days, *p* = 0.02) without major differences in postoperative complications [39]. A 2018 meta-analysis of 11 randomized controlled trials comparing FC-SEMS, PC-SEMS, and U-SEMS found a non-significant 32% reduction in both stent failure and mortality in the C-SEMS group, with no differences in adverse event rates [32]. More recent studies have shown a significantly longer time to recurrent biliary obstruction with C-SEMS [40], whereas Vanella et al. reported a shorter time to recurrent biliary obstruction with FC-SEMS compared with PC-SEMS [33].

A comprehensive systematic review and meta-analysis published in June 2025, comprising 21 studies and 5753 patients, revealed that C-SEMS demonstrated significantly higher post-ERCP acute cholecystitis rates, substantially lower tumor ingrowth rates, and markedly higher stent migration rates compared to alternatives. Notably, this study performed the first sub-analysis based on gallbladder presence, with findings significant for both groups [41]. Table 1 provides a comprehensive comparison of different stent types.

### 2.2. TBD in Resectable Distal Malignant Biliary Obstruction

The American College of Gastroenterology (ACG) 2023 guidelines recommend against preoperative TBD in most resectable DMBO cases. Notable exceptions include acute cholangitis, severe pruritus, markedly elevated serum bilirubin, and patients undergoing neoadjuvant therapy or experiencing anticipated surgical delays [12,14]. A meta-analysis of 25 studies (3 RCTs and 22 retrospective studies) demonstrated that preoperative biliary drainage was associated with significantly higher overall complications and a nearly 2-fold increase in wound infections, without meaningful differences in mortality, pancreatic fistula, or intra-abdominal abscess formation. Notably, this analysis did not stratify outcomes by stent type [42].

A landmark multicenter RCT in NEJM by van der Gaag et al. (2010) demonstrated that routine preoperative biliary drainage in patients with resectable pancreatic head cancer nearly doubled the rate of serious complications compared to surgery alone (74% vs. 39%), despite successful drainage in 94% of patients [43]. This trial established the evidence base for current guidelines recommending against routine preoperative drainage in resectable disease, reserving it for specific indications such as acute cholangitis, severe pruritus, or patients requiring neoadjuvant therapy [43].

For borderline resectable tumors, ACG guidelines specifically recommend SEMS due to significantly longer patency and reduced cholangitis events, minimizing interruptions to neoadjuvant or palliative chemotherapy. Lower reintervention rates and reduced stent-related hospitalizations contribute to improved general and disease-specific quality of life compared with plastic stents [12,23,25,44]. For surgical DMBO candidates, FC-SEMS is recommended over U-SEMS due to ease of removal prior to surgery. However, if U-SEMS placement occurs in potential pancreaticoduodenectomy candidates, the proximal end should be positioned at least 1.5 cm below the biliary confluence to ensure adequate healthy duct length for biliary-enteric anastomosis [45,46,47].

### 2.3. TBD in Unresectable Hilar Malignant Biliary Obstruction

Patients with HMBO typically present at an unresectable stage, precluding surgical intervention in most cases. Specific surgical contraindications include severe medical comorbidities, distant metastases, major vascular structure involvement not amenable to reconstruction, bilateral segmental ductal involvement, unilateral segmental ductal extension with contralateral vascular involvement, and inadequate future remnant liver volume. Instead, palliative endoscopic TBD via ERCP and PTBD represent the most commonly employed approaches [48,49].

The Bismuth–Corlette classification guides HMBO management. Type 1 and 2 obstructions are typically managed similarly to DMBO, utilizing endoscopic TBD [14]. However, management of type 3 and 4 disease remains less clearly defined. Asian-Pacific consensus and European Society of Gastrointestinal Endoscopy (ESGE) guidelines recommend PTBD or combined PTBD-ERCP rather than endoscopic drainage alone for high-level stenosis (Bismuth type III or IV), considering technical difficulties and complication risks [14,50]. ACG guidelines adopt a more individualized approach, suggesting that selection should be based on disease characteristics, patient factors (comorbidities, life expectancy, anesthesia tolerance), local expertise availability, and patient preferences [12].

Optimal drainage approach selection for HMBO requires careful consideration of multiple factors, including technical feasibility, patient suitability, technique durability, and complication rates [51]. Paik et al. demonstrated superior technical success for percutaneous SEMS compared with endoscopic SEMS (92.7% vs. 77.3%, *p* < 0.05) for high-grade hilar cholangiocarcinoma, with similar complications, stent patency, and survival [52,53].

However, PTBD complications, including external catheter-related pain and discomfort, technical difficulties with ascites, liver metastases, or coagulopathy, non-dilated biliary systems, and risks of tube dislodgement or peri-catheter leak have led to increasing preference for endoscopic palliation. Additionally, endoscopic TBD utilizes physiological drainage pathways, offering greater convenience compared with PTBD.

For advanced HMBO, debate continues, with Asia-Pacific and ESGE guidelines favoring PTBD, while ASGE guidelines suggest individualized decisions based on patient preference, local expertise, and disease characteristics [14,49,53]. Importantly, these approaches should be viewed as complementary rather than competing modalities.

Drainage of at least 50% of liver volume represents a crucial target, as this threshold significantly predicts drainage effectiveness, correlating with lower cholangitis risk and longer patient survival in HMBO [54]. Achieving this typically requires bilateral stenting, though debate persists regarding unilateral versus bilateral approaches. While unilateral drainage offers technical simplicity and lower adverse event rates, guidelines recommend multiple stent placement to achieve >50% liver volume drainage in advanced HMBO due to incomplete drainage concerns [55,56,57,58].

Multiple stent placement presents greater technical challenges and may increase adverse outcomes such as acute cholangitis. Risk mitigation strategies include pre-procedural 3D-CT/MRCP for detailed image analysis and performance by experienced endoscopists at high-volume tertiary centers. A 2019 systematic review and meta-analysis of 9 studies with 782 patients comparing unilateral versus bilateral stenting for inoperable high-grade biliary strictures reported approximately 40% lower reintervention rates for bilateral stenting, without differences in technical success, early complications, late complications, or stent malfunction [57].

Regarding stent type for hilar obstruction, guidelines favor U-SEMS due to superior long-term drainage, reduced reintervention requirements, and preservation of cystic and intrahepatic duct outflow. Plastic stents retain utility when initial decompression is required before definitive drainage planning [12]. A February 2025 systematic review and meta-analysis of seven studies reported excellent technical and clinical success rates for C-SEMS (>90% for both). Overall adverse events occurred in approximately one-sixth of cases, predominantly cholangitis, pancreatitis, liver abscess, and cholecystitis. While stent migration was relatively uncommon, recurrent biliary obstruction developed in nearly half of patients at a median of 142 days, though reintervention was highly successful in over 90% of cases [58].

When performing bilateral stenting, the choice between side-by-side (SBS) and stent-in-stent (SIS) techniques requires consideration. A 2022 meta-analysis found that while SIS demonstrated more favorable technical success and early complication rates, SBS achieved longer stent patency [59]. However, a more recent 2025 retrospective analysis of 62 HMBO patients reported shorter stent patency in the SBS group compared with SIS (147 days vs. 252 days to recurrent biliary obstruction) [60].

### 2.4. TBD in Resectable Hilar Malignant Biliary Obstruction

Preoperative TBD is generally not recommended for resectable HMBO, with notable exceptions including low future liver remnant (FLR) volume (<30%), cholangitis, intractable pruritus, hyperbilirubinemia-induced malnutrition, hepatic or renal insufficiency, prior to neoadjuvant therapy, and anticipated surgical delays [48]. In cases of inadequate FLR, portal vein embolization and preoperative TBD are required to achieve remnant liver hypertrophy and decrease hepatic insufficiency risk, respectively [61]. A 2017 meta-analysis of retrospective studies found preoperative TBD associated with higher post-surgical infectious complications without survival benefit (40% vs. 17%), potentially due to biliary microbiome shifts promoting resistant bacteria [62].

No consensus exists regarding the optimal drainage technique for patients requiring preoperative drainage. Two meta-analyses comparing endoscopic TBD and PTBD reported lower cholangitis risk with PTBD, particularly in Bismuth III/IV disease. However, a randomized controlled trial comparing these approaches was prematurely terminated due to higher mortality in the PTBD group (41% vs. 11%, *p* = 0.03) [62,63,64]. Although some studies favor nasobiliary drainage over endoscopic stenting and PTBD, its use is limited by prolonged hospitalization, the risk of inadvertent self-removal, and patient discomfort [65,66].

Bilirubin > 3 mg/dL following preoperative biliary drainage serves as a negative survival indicator. Furthermore, delaying surgery beyond 2 weeks after drainage has been associated with bacterial translocation and tumor dissemination. Therefore, surgery should proceed promptly after achieving target bilirubin levels, with prophylactic antibiotics administered [67].

### 2.5. Endoscopic Ultrasound-Guided Biliary Drainage

Although ERCP has served as the mainstay for therapeutic MBO management, endoscopic ultrasound-guided biliary drainage (EUS-BD) has gained substantial popularity over the past two decades due to its ability to bypass tumor invasion and avoid pancreatic duct instrumentation.

A meta-analysis of six randomized controlled trials involving 570 patients compared EUS-BD with ERCP, demonstrating comparable outcomes between the two approaches. No significant differences were observed in stent patency duration, procedural efficiency, patient survival, technical or clinical success rates, or safety profile, including overall adverse events and cholangitis. However, EUS-BD demonstrated significant clinical advantages, including approximately one day shorter hospital stays and substantially lower risks of reintervention (nearly 40% reduction), post-procedural pancreatitis (85% reduction), and tumor ingrowth/overgrowth (72% reduction) [68,69].

A more recent 2025 systematic review and meta-analysis comparing EUS-BD to ERCP (339 vs. 331 patients) across eight studies confirmed these findings, demonstrating that EUS-BD significantly reduced stent dysfunction risk by more than half, decreased post-procedure pancreatitis by approximately 75%, and reduced tumor ingrowth or overgrowth by 73%. Additionally, EUS-BD showed substantially lower adverse event risk compared with PTBD (63% reduction) and reduced hospital stay by approximately one day compared with both ERCP and PTBD [70]. Table 2 provides a comprehensive comparison of EUS-BD techniques.

### 2.6. Endoscopic Ultrasound-Guided Choledochoduodenostomy

EUS-guided choledochoduodenostomy (EUS-CDS) establishes a direct tract between the duodenum and the common bile duct, thereby bypassing tumor-related biliary obstruction. While the procedure has existed for years, it has been substantially simplified by lumen-apposing metal stents (LAMS) compared with plastic stents or SEMS. SEMS were initially preferred over plastic stents due to higher peritonitis and occlusion risks with plastic stents, resulting in cholangitis and repeat interventions. Furthermore, SEMS offered increased drainage diameter and stent patency [71,72]. Following LAMS introduction, multiple meta-analyses have compared these stent types. A 2021 meta-analysis including 31 studies and 820 patients who underwent EUS-CDS with either SEMS or LAMS demonstrated comparable technical and clinical success rates and adverse event profiles, although LAMS deployment was associated with shorter procedure times [72]. In contrast, a more recent December 2024 meta-analysis including 6 RCTs and 583 patients concluded that EUS-CDS with LAMS demonstrated significantly higher technical success compared with both EUS-CDS with SEMS (approximately 20% improvement) and ERCP (approximately 17% improvement) [73].

Multiple meta-analyses assessing EUS-CDS have reported high technical success rates (93.5–96%) and clinical success rates (88–96%), with overall complication rates ranging from 5.2% to 20% [72,74,75,76,77,78]. The most common adverse events are cholangitis and cholecystitis, while less frequent complications include, but are not limited to, peritonitis, bleeding, bilioperitoneum, pneumoperitoneum, abdominal pain, stent migration, and double mucosal puncture [79]. A meta-analysis reported that EUS-CDS was associated with a 14% overall complication rate, including cholangitis, bleeding, bile leak, and perforation [80]. Despite high success rates, understanding factors determining success is crucial. A 2025 retrospective analysis of 296 patients across 23 centers identified multivariate risk factors for technical failure, including CBD diameter ≤ 15 mm, duodenal stenosis, wired technique, and low operator experience (≤10 LAMS procedures) [81]. Another multivariate analysis of two RCTs with 152 patients similarly found extrahepatic bile duct diameter ≤ 15 mm associated with higher stent misdeployment and technical failure risk [82].

Stent misdeployment represents an important complication requiring prompt management. Beunon et al. reported successful endoscopic rescue in 53% of patients using guidewire-assisted FC-SEMS placement and in 22% through placement of a new LAMS. Additional salvage strategies included ERCP, EUS-guided hepaticogastrostomy, and gallbladder drainage. Overall, endoscopic rescue was successful in 77% of cases and was associated with fewer severe complications, although 12% of technical failures were still accompanied by 30-day mortality [81].

EUS-CDS dysfunction, a long-term complication characterized by bilirubin rise or cholangitis onset, leads to impaired biliary drainage requiring reintervention, with reported rates ranging from 2.7 to 55% [71]. Vanella et al. identified duodenal invasion as an independent predictor of LAMS dysfunction and proposed a five-type classification system for stent dysfunction: stone impaction (33.3%), food impaction (18.5%), LAMS invasion or compression on the duodenal side (11.1%), and other types [83]. Key management strategies include coaxial double-pigtail stent insertion through the LAMS, balloon-assisted stent lumen clearance, and downstream gastrointestinal obstruction treatment without direct LAMS manipulation. Endoscopic reintervention effectively restores patency in most cases, with percutaneous or surgical drainage reserved for refractory situations. New EUS-CDS or PTBD placement may be feasible when needed. Additionally, the existing LAMS may serve as an access route for guidewire advancement for antegrade SEMS deployment or facilitating a rendezvous approach [71].

Given the high morbidity and mortality associated with stent dysfunction, prevention strategies merit consideration. The two most reported approaches include routine placement of either a coaxial double-pigtail stent or a covered SEMS within the LAMS. The double-pigtail stent functions as a spacer, preventing opposite bile duct wall collapse against the LAMS and subsequent lumen obstruction. A covered SEMS provides similar protection while directing biliary flow toward the duodenal anastomosis side, encouraging drainage alongside the LAMS rather than through its lumen, thereby minimizing clogging and reflux [71].

### 2.7. Endoscopic Ultrasound-Guided Hepaticogastrostomy

EUS-guided hepaticogastrostomy (EUS-HGS) was first described by Giovannini et al. in 2003 and represents a palliative biliary drainage technique for unresectable hilar obstruction in which EUS guidance is used to create a fistulous tract between the dilated left hepatic duct and the stomach [84,85]. EUS-HGS provides an important alternative for biliary drainage in malignant biliary obstruction when ERCP is unsuccessful or not feasible. Contraindications include significant ascites, hepatic masses precluding safe biliary access, and intervening vascular structures, particularly in the setting of portal hypertension [85,86].

Current ESGE guidelines recommend EUS-CDS over EUS-HGS for DMBO management due to lower adverse event rates [14,80]. EUS-guided hepaticogastrostomy (EUS-HGS) may be preferred when duodenal access is not feasible, including scenarios of gastric outlet obstruction or surgically altered anatomy, in which an intrahepatic EUS-HGS approach serves as the optimal alternative [85].

A 2024 meta-analysis including nine non-randomized studies and two randomized controlled trials, encompassing 537 patients undergoing either EUS-HGS or EUS-CDS, assessed technical and clinical success, adverse events, and procedure duration. Technical and clinical success rates were comparable between the two techniques, while adverse events were significantly higher in the EUS-HGS group. Mean procedure times did not differ significantly between approaches [87].

A study examining EUS-HGS long-term effects found a median overall survival of 144 days post-procedure. Adverse events occurred during follow-up in 65 patients (33%). Multivariate analysis revealed PC-SEMS use was associated with significantly lower recurrent biliary obstruction risk, with approximately 50% reduction in hazard. Additionally, patients with distal stenoses demonstrated better stent patency. Recurrent biliary obstruction developed in 38 cases (19.1%), with tumor ingrowth causing 36.8% of these. Other major adverse effects included abdominal pain (12.1%), infection (18.3%), peritonitis (5.6%), bleeding (6.1%), cholangitis (4.5%), biloma (3.2%), and cholecystitis (1.1%) [88]. This study notably represented the largest cohort examining EUS-HGS’s longer-term impact rather than early adverse event rates such as stent migration or bile leakage.

### 2.8. EUS-Guided Rendezvous

EUS-guided rendezvous (EUS-RV), also termed EUS-assisted bile duct access, represents a technically demanding rescue technique performed after failed ERCP. It provides an alternative approach, allowing transpapillary stenting via ERCP over a guidewire placed through prior EUS-guided intrahepatic or extrahepatic biliary access. Technical success rates range between 72 and 96%, with a mean of 84–86% in expert hands. However, adverse events occur in 10–34% of cases. Current ESGE guidance restricts its indication mainly to benign biliary disease with normal anatomy after a second failed ERCP. A recent retrospective observational study found EUS-RV for malignant disease associated with worse outcomes and higher adverse effects compared with benign pathology [89].

### 2.9. EUS-Guided Antegrade Drainage

EUS-guided antegrade drainage (EUS-AG) involves biliary access with guidewire advancement through the stricture into the small bowel lumen, followed by antegrade metal stent deployment through the papilla or anastomosis in altered anatomy. Though technically difficult with high failure rates, this technique preserves normal anatomy. A recent systematic review analyzing nine studies with a total of 210 patients found that the procedure achieved technical success in 92% of cases, with complications occurring in 14% of patients, the most common being post-procedural pancreatitis [14,90].

### 2.10. EUS-Guided Gallbladder Drainage

EUS-guided gallbladder drainage (EUS-GBD), long utilized for cholecystitis treatment, has recently gained popularity for biliary drainage when ERCP is contraindicated. Three recent meta-analyses evaluating EUS-GBD’s role in DMBO reported clinical success rates of 85–89%, adverse events in 10–13%, technical success of 99.2–100%, and dysfunction rates of 9% [91,92,93]. Most included studies were retrospective from expert tertiary centers, potentially skewing results favorably toward EUS-GBD.

The multicenter GALLBLADEUS study compared EUS-guided choledochoduodenostomy with EUS-guided gallbladder drainage in 37 and 41 patients, respectively. Clinical and technical success were similar between the groups, but late complications beyond 24 h were more frequent with EUS-CDS, occurring in 21.6 percent of patients versus 7.3 percent with EUS-GBD [94].

## 3. Emerging Developments and Future Directions

### 3.1. EUS-Guided Gastroenterostomy Versus Surgical Gastrojejunostomy

The current gold standard treatment for gastric outlet obstruction (GOO) involves either surgical gastroenterostomy or endoscopic stenting (ES). Surgery carries invasiveness with high adverse event rates, while ES demonstrates high reintervention rates and stent patency limitations. EUS-guided gastroenterostomy (EUS-GE) has emerged as an alternative, potentially providing longer stent patency without surgical invasiveness [95,96].

A systematic review and meta-analysis of 5 studies (507 patients), including 1 RCT and 4 matched-control studies, reported that EUS-GE demonstrated nearly 3-fold higher odds of clinical success compared to ES. The study concluded EUS-GE represents a reasonable option for malignant GOO treatment with high success rates, particularly at tertiary centers [95].

The ENDURO trial, a multicenter RCT published in The Lancet in December 2025, compared endoscopic versus surgical gastroenterostomy for malignant GOO palliation in 98 patients (48 endoscopic, 50 surgical). The endoscopic group demonstrated a shorter time to solid oral intake (median 1 vs. 3 days). Both groups had comparable persistent or recurrent obstructive symptoms requiring reintervention (5 vs. 6 patients). Overall adverse events occurred in 28 (58%) endoscopic patients versus 32 (64%) surgical patients (RR 0.91, 95% CI 0.66–1.25). Three fatal events occurred in the surgical group compared with one in the endoscopic group [96]. These promising results in both clinical outcomes and cost-effectiveness warrant additional randomized studies.

### 3.2. Artificial Intelligence in Predicting Stent Failure and Complications

Artificial intelligence (AI) use in gastroenterology has expanded rapidly in recent years. For MBO, emerging studies have explored the AI-based model’s ability to predict outcomes such as stent failure and complications, including cholangitis. Earlier and more accurate high-risk scenario identification may support future targeted preventive strategies.

A retrospective study of 218 patients investigated how machine learning could inform cholangitis risk factors post-ERCP stent implantation. Twenty-seven clinical variables served as input for seven models subsequently trained and tested for classification prediction. The Reinforcement Fine-Tuning (RFT) model achieved high success with reported accuracies up to 0.86 and area under the receiver operator characteristic curve (AUROC) up to 0.87 [97].

A comparative analysis of 285 patients published in 2025 compared logistic regression and artificial neural network (ANN) models in predicting early mortality following stent placement in MBO. The logistic regression model using CA19-9 and prior ERCP as key predictors achieved moderate discriminative performance (AUC 0.727, accuracy 65%). In contrast, the ANN model incorporating five clinical variables demonstrated superior overall performance (AUC 0.813, accuracy 88.2%), with notably higher specificity (95.5% vs. 83.3%), but marginally lower sensitivity (61.1% vs. 61.2%) [98].

A retrospective multicenter study examining AI’s ability to predict post-ERCP cholangitis (PEC) in MBO patients selected radiofrequency ablation, white blood cell count, jaundice severity, and serum amylase as independent PEC risk factors. These were then input into six machine learning models and tested on 395 patients. Among the six models, XGBoost performed best on external patients with an AUC of 0.7270 [99].

These studies show promise that AI may eventually help predict complications in high-risk patients, allowing early individualized treatment planning and hopefully preventing these complications. Further development and testing with larger cohorts remains necessary.

## 4. Conclusions

**Figure 1:**
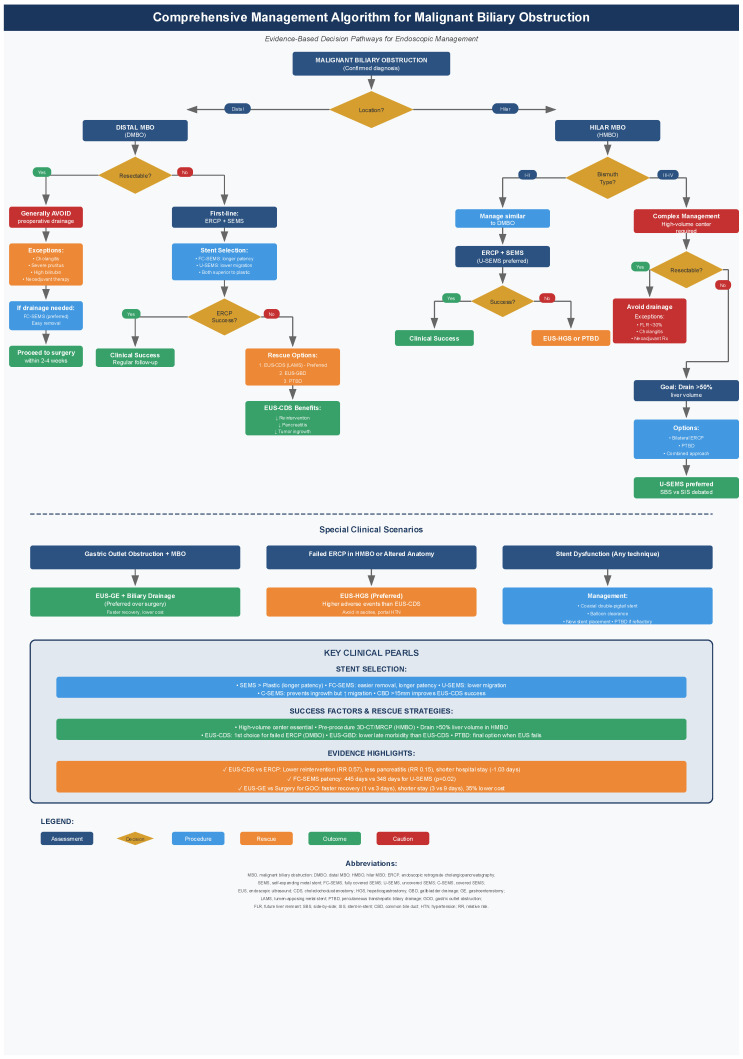
Comprehensive Management Algorithm for Malignant Biliary Obstruction. Decision-making flowchart for endoscopic intervention selection based on anatomical location, resectability status, and technical feasibility.

Malignant biliary obstruction requires timely expert intervention to prevent severe morbidity and mortality [1,2,3,4,5]. The therapeutic landscape has evolved significantly, with ERCP and EUS-guided approaches now serving as complementary modalities in the management algorithm presented in Figure 1. While ERCP with SEMS placement remains the gold standard for most cases [12,13,14], the evidence now supports EUS-guided biliary drainage as a valuable alternative offering reduced reintervention rates, lower pancreatitis risk, and shorter hospitalizations [67,68,69].

The optimal therapeutic approach varies by anatomical location and clinical context. For distal MBO, FC-SEMS demonstrates superior patency compared to U-SEMS (445 vs. 348 days) [39], though migration risk remains a consideration [32,33,34,40,41]. In hilar MBO, achieving ≥ 50% liver volume drainage through bilateral stenting when feasible is crucial for optimal outcomes [53], though technical complexity must be balanced against complication risk [56,57,58,59]. EUS-guided techniques offer distinct advantages in specific scenarios: EUS-CDS achieves 93.5–96% technical success for DMBO with lower tumor ingrowth [71,72,73,74,75,76,77], EUS-GBD demonstrates lower late morbidity (7.3% vs. 21.6%) [93], and EUS-HGS serves specific roles in HMBO and altered anatomy, though with higher adverse events [84,85,86,87]. For concurrent malignant gastric outlet obstruction, EUS-GE provides faster recovery, shorter hospitalization, and lower costs versus surgery [95,96].

These therapeutic strategies align with the current international consensus. Recent European Society of Gastrointestinal Endoscopy (ESGE) guidelines on the diagnostic work-up of biliary strictures emphasize the multimodal approach incorporating both ERCP-based and EUS-guided sampling techniques tailored to stricture location and clinical context [100]. Similarly, the World Endoscopy Organization (WEO) guidelines on ERCP biliary cannulation and sphincterotomy techniques discuss optimized cannulation, alternate access techniques for challenging cases, and strategies to prevent complications like post-ERCP pancreatitis [101]. Together, these guidelines published within the last year reinforce the diagnostic and therapeutic principles discussed in this review.

Optimal outcomes require high-volume tertiary centers with experienced endoscopists [12,14]. Treatment selection must be individualized based on tumor characteristics (distal vs. hilar, resectable vs. unresectable), anatomical considerations, patient factors, and local expertise, as outlined in Table 3 [12,47,48,49]. Future priorities include head-to-head trials comparing EUS-BD with ERCP as first-line therapy, standardization of EUS-BD techniques, validation of AI-based predictive models for complication risk [97,98,99], and development of novel stent designs to improve patency and reduce dysfunction; key recent studies informing these priorities are summarized in Table 4.

## Figures and Tables

**Table 1:** Comparison of Stent Types for Malignant Distal Biliary Obstruction.

Stent Type	Advantages	Disadvantages	Clinical Recommendations
Plastic Stents [22,23,24,25,26]	Lower cost Easy removal Lower pancreatitis risk	Shorter patency Frequent reinterventions Higher occlusion rates	Limited role; consider for temporary drainage or very short life expectancy
Uncovered SEMS [32,33,34,35,36,37,38]	Longer patency vs. plastic Lower migration risk Good tissue integration	Tumor ingrowth Difficult removal Not ideal for resectable disease	Suitable for unresectable DMBO; preferred for hilar obstruction
Covered SEMS (FC/PC) [32,33,34,35,36,37,38]	Prevents tumor ingrowth Easy removal (FC-SEMS) Longer patency in some studies	Higher migration risk Tumor overgrowth Higher cholecystitis risk Sludge formation	FC-SEMS preferred for resectable/borderline resectable disease; consider for unresectable DMBO

**Table 2:** Comparison of EUS-Guided Biliary Drainage Techniques.

Technique	Primary Indication	Technical Success	Clinical Success	Major Complications
EUS-CDS [71,72,73,74,75,76,77,78,79,80,81,82,83]	Failed ERCP in DMBO; preferred EUS-BD approach for distal obstruction	93.5–96%	88–96%	Cholangitis (4%) Bleeding (4%) Bile leak (4%) Stent dysfunction (2.7–55%)
EUS-HGS [84,85,86,87,88]	Failed ERCP in HMBO; gastric outlet obstruction; altered anatomy	Similar to EUS-CDS	Similar to EUS-CDS	Higher adverse events vs. EUS-CDS (OR 2.01) Infection (18.3%) Abdominal pain (12.1%) Bleeding (6.1%)
EUS-RV [89]	Failed ERCP (second attempt); benign biliary disease with normal anatomy	72–96% (expert hands)	84–86%	10–34% adverse events Worse outcomes in malignant disease
EUS-AG [14,90]	Failed ERCP with preservation of normal anatomy	92%	Not reported	14% adverse events Pancreatitis (predominant)
EUS-GBD [91,92,93,94]	Failed ERCP in DMBO; alternative to EUS-CDS with potentially lower late morbidity	99.2–100%	85–89%	10–13% adverse events 9% dysfunction rate Lower late morbidity vs. EUS-CDS (7.3% vs. 21.6%)

Abbreviations: EUS-CDS, endoscopic ultrasound-guided choledochoduodenostomy; EUS-HGS, endoscopic ultrasound-guided hepaticogastrostomy; EUS-RV, endoscopic ultrasound-guided rendezvous; EUS-AG, endoscopic ultrasound-guided antegrade; EUS-GBD, endoscopic ultrasound-guided gallbladder drainage; DMBO, distal malignant biliary obstruction; HMBO, hilar malignant biliary obstruction; ERCP, endoscopic retrograde cholangiopancreatography.

**Table 3:** Clinical Decision-Making Algorithm for Endoscopic Management of Malignant Biliary Obstruction.

Clinical Scenario	First-Line Approach	Second-Line/Rescue	Key Considerations
Unresectable DMBO [12,14,23,24,25,32,33,39,40,41]	ERCP with FC-SEMS or U-SEMS	EUS-CDS with LAMS or EUS-GBD	SEMS preferred; C-SEMS for longer patency
Resectable/Borderline Resectable DMBO [12,14,42,43,44,45,46,47]	SEMS (FC-SEMS preferred)	Avoid preoperative drainage except: cholangitis, high bilirubin, neoadjuvant therapy	FC-SEMS allows easy removal; Place ≥ 1.5 cm below confluence
Unresectable HMBO (Bismuth I–II) [12,14]	ERCP with SEMS (U-SEMS preferred)	EUS-HGS or PTBD	Similar to DMBO management
Unresectable HMBO (Bismuth III–IV) [12,14,48,49,50,51,52,53,54,55,56,57,58,59,60]	Bilateral ERCP stenting (>50% liver volume) OR PTBD	EUS-HGS or Combined approach	High-volume center; 3D-CT/MRCP; U-SEMS preferred
Resectable HMBO [48,61,62,63,64,65,66,67]	Generally avoid drainage; If needed: ERCP or PTBD	Surgery within 2 weeks after target bilirubin	Indications: FLR < 30%, cholangitis, high bilirubin, neoadjuvant therapy
Failed ERCP in DMBO [68,69,70,71,72,73,80,81,82,83]	EUS-CDS with LAMS OR EUS-GBD	PTBD	Lower reintervention, pancreatitis; CBD > 15 mm better; EUS-GBD lower late morbidity
Failed ERCP in HMBO [80,84,85,86,87,88]	EUS-HGS	PTBD	Higher adverse events; Avoid in ascites, portal hypertension; PC-SEMS reduces RBO
Gastric Outlet Obstruction + MBO [96]	EUS-GE with biliary drainage	Surgical gastrojejunostomy	Faster recovery (1 vs. 3 days), shorter stay (3 vs. 9 days), lower cost

Abbreviations: DMBO, distal malignant biliary obstruction; HMBO, hilar malignant biliary obstruction; ERCP, endoscopic retrograde cholangiopancreatography; SEMS, self-expanding metal stent; FC-SEMS, fully covered SEMS; U-SEMS, uncovered SEMS; C-SEMS, covered SEMS; EUS-CDS, endoscopic ultrasound-guided choledochoduodenostomy; EUS-GBD, endoscopic ultrasound-guided gallbladder drainage; EUS-HGS, endoscopic ultrasound-guided hepaticogastrostomy; LAMS, lumen-apposing metal stent; PTBD, percutaneous transhepatic biliary drainage; FLR, future liver remnant; EUS-GE, endoscopic ultrasound-guided gastroenterostomy; RBO, recurrent biliary obstruction; CBD, common bile duct.

**Table 4:** A summary of the top five papers over the past two years regarding MBO.

Authors, Year	Title	Type of Study	Number of Studies/Patients	Key Findings
Lopimpisuth et al., 2025 [41]	Postprocedural cholecystitis following covered self-expandable metal stent placement in patients with distal malignant biliary obstruction	Systematic Review and Meta-Analysis	21 studies 5753 patients	CSEMS showed higher post-ERCP acute cholecystitis rates, lower tumor growth rates, and higher rates of stent migration
Chung et al., 2025 [58]	Efficacy and safety of covered self-expandable metal stents for malignant hilar biliary obstruction	Systematic Review and Meta-Analysis	7 studies 194 patients	High technical and clinical success rates of CSEMS placement in MHBO. Adverse events: cholangitis, cholecystitis, and pancreatitis were <10%.
Zafar et al., 2025 [70]	Efficacy of endoscopic ultrasound-guided biliary drainage of malignant biliary obstruction	Systematic Review and Meta-Analysis	8 studies 670 patients	EUS-BD performed better than ERCP-BD and PTBD in reducing stent dysfunction, postprocedural pancreatitis, and tumor ingrowth or overgrowth.
Lauri et al., 2024 [73]	Primary drainage of distal malignant biliary obstruction	A comparative network meta-analysis	6 RCTs 583 patients	EUS-CDS with LAMS had the highest technical and clinical success rates and was significantly superior to ERCP as the upfront technique for dMBO treatment.
van de Pavert et al., 2025 [96]	Endoscopic versus surgical gastroenterostomy for palliation of malignant gastric outlet obstruction (ENDURO)	Multicenter RCT	98 patients (48 endoscopic, 50 surgical)	Endoscopic group demonstrated shorter time to solid oral intake (1 vs. 3 days). Comparable reintervention rates (5 vs. 6 patients). Overall adverse events 58% endoscopic vs. 64% surgical (RR 0.91, 95% CI 0.66–1.25). Three fatal events in surgical group vs. one in endoscopic group.

## Data Availability

No new data were created or analyzed in this study. Data sharing is not applicable to this article.
